# The Genetic Puzzle of the Stress-Induced Cardiomyopathy (Takotsubo Syndrome): State of Art and Future Perspectives

**DOI:** 10.3390/biom15070926

**Published:** 2025-06-24

**Authors:** Domenico Lio, Letizia Scola, Giusi Irma Forte, Loredana Vaccarino, Manuela Bova, Patrizia Di Gangi, Giorgia Santini, Daniela di Lisi, Cristina Madaudo, Giuseppina Novo

**Affiliations:** 1 University Research Center “Migrate”, University of Palermo, 90100 Palermo, Italy; 2 Clinical Pathology, Department of Bio-Medicine, Neuroscience and Advanced Diagnostics, University of Palermo, 90133 Palermo, Italy; letizia.scola@unipa.it (L.S.); loredanavaccarino@libero.it (L.V.); 3 Institute of Molecular Bioimaging and Physiology (IBFM), National Research Council (CNR), Cefalù Secondary Site, C/da Pietrapollastra-Pisciotto, 90015 Cefalù, Italy; giusi.forte@ibfm.cnr.it; 4 Clinical Pathology, Territorial Laboratories, ASP Palermo, 90141 Palermo, Italy; manuela.bova@asppalermo.org (M.B.); patrizia.digangi@asppalermo.org (P.D.G.); 5 Clinical Pathology Unit, Buccheri La Ferla Hospital of Palermo, 90123 Palermo, Italy; santini.giorgia@fbfpa.it (G.S.); daniela.dilisi@policlinico.pa.it (D.d.L.); 6 Cardiology Unit, University Hospital “Paolo Giaccone”, 90127 Palermo, Italy; cristina.madaudo@unipa.it (C.M.); giuseppina.novo@unipa.it (G.N.); 7 Department of Health Promotion, Mother and Child Care, Internal Medicine and Medical Specialties (ProMISE), University of Palermo, 90133 Palermo, Italy

**Keywords:** takostubo syndrome, stress-induced cardiomyopathy, genetics, genetic polymorphisms, genetic markers, GWAS, full exome sequencing, miR, inflammation

## Abstract

Takotsubo syndrome (TS), also known as stress-induced cardiomyopathy, is classically characterized by an acute onset mimicking myocardial infarction and by distinctive transient wall motion abnormalities detectable via echocardiography, often resembling a Japanese octopus trap (the so-called “takotsubo”). The possibility that a genetic background may contribute to TS susceptibility emerged early, supported by several familial case reports. Despite a large number of investigations, no definitive genetic markers associated with TS risk have been conclusively identified. The lack of a clear Mendelian inheritance pattern suggests a multifactorial etiology and pathogenesis, likely involving complex gene–environment interactions and a polygenic background. This review analyzes the genetic variants implicated in the different functional pathways contributing to TS pathogenesis and discusses the current state of knowledge regarding its genetic underpinnings. Finally, we propose future directions for research aimed at identifying a multigene susceptibility panel that could be useful in diagnosis, prevention strategies, and the identification of novel therapeutic targets for individuals at high risk. We conclude that innovative approaches based on data-mining algorithms and nonlinear analytic methods applied to large patient datasets may be instrumental in resolving the genetic complexity of TS.

## 1. Introduction

Takostubo syndrome (TS) is classically characterized by an acute onset mimicking myocardial infarction and peculiar transient wall motion abnormalities that can be detected by echocardiographic imaging, suggestive of a Japanese octopus or fish trap (the so called takotsubo). These abnormalities often involve apical and mid-ventricular akinesia with hyperkinesia of the basal segments, resulting in the typical “apical ballooning” pattern [1]. Variants of this presentation have also been described [1]. The syndrome predominantly affects postmenopausal women (accounting for 85–90% of cases), often in the context of an intense emotional or physical stressor [2].

Epidemiological data confirm a marked sex disparity, with 80–90% of cases occurring in women over 55 years old [3,4]. However, a recent age-stratified analysis revealed a higher incidence among younger men, who also exhibit a threefold increase in mortality and major complications compared to women [5].

Generally, acute symptoms recede, and patients return to an apparent healthy status. This reversible nature allowed us, for a long time, to consider TS a benign disease. In recent years, systematic registries and long-term follow-up studies have revealed that TS patients carry risks similar to those of acute myocardial infarction (AMI), including arrhythmias, cardiogenic shock, thromboembolic events, ventricular rupture, and acute heart failure [6]. In the largest TS registries, mortality exceeds 5%, with cardiogenic shock, malignant arrhythmias, ventricular rupture, and ischemic stroke among the primary causes of death [7,8].

Since the initial diagnostic criteria proposed by the Mayo Clinic [9], the understanding and classification of TS have evolved. The European Society of Cardiology has recently included TS in the Fourth Universal Definition of Myocardial Infarction and developed a diagnostic algorithm assigning a probability score based on female sex prevalence, presence of stressors, absence of STEMI signs, and associated psychiatric or neurological conditions. TS may also be accompanied by moderately elevated cardiac biomarkers (troponin, BNP), and the presence of coronary artery disease does not exclude its diagnosis, although infectious myocarditis remains an exclusion criterion [4,7,10].

Emerging evidence supports the existence of two main clinical forms of TS: primary TS, occurring in previously healthy individuals presenting with acute cardiac symptoms, and secondary TS, developing in patients with underlying physical or psychiatric conditions or following medical interventions (e.g., surgery, trauma, subarachnoid hemorrhage, stroke, endocrine tumors) [11]. Finally, TS may also lead to persistent subclinical myocardial dysfunction and residual metabolic, structural, or functional abnormalities [8]. Complications such as ventricular thrombus formation and ischemic stroke are not uncommon [4].

The pathogenesis of TS is multifactorial and not yet fully elucidated. Proposed mechanisms include catecholamine excess, coronary microvascular dysfunction or vasospasm, autonomic imbalance, calcium handling abnormalities, basal hypercontractility with outflow tract obstruction, estrogen deficiency, and systemic or local inflammation [4,7,12,13].

One of the most consistent findings is the role of excessive catecholaminergic stimulation, particularly involving β2-adrenergic receptor activation, which leads to myocardial stunning, transient systolic dysfunction, and regional wall motion abnormalities, with an acute but generally reversible reduction in left ventricle mid-chamber functionality [7]. Catecholamines act on cardiomyocytes via adrenergic receptor (AR) β1ARs and β2ARs that lead to the increment of contractility. Engaged β1ARs and β2ARs bound the intracellular G-protein-activated (Gαs) proteins that activate adenylyl cyclase and in turn cAMP production and contractility increase [14]. This effect is then physiologically counteracted in a continuous dynamic equilibrium by inhibitory G-protein (Gαi) proteins that depress cardiac contractility [7]. In TS patients, cardiac sympathetic activity is increased with the exception of the akinetic regions of the left ventricle apex when compared to base hyper-contracting myocardium. Binding of supra-physiological epinephrine, concentrations lead to an excessive activation of Gαi, modifying intracellular balance between stimulation and inhibitory signals and in turn facilitating the apical ballooning appearance [15,16,17].

Supporting this model are cases of TS triggered by pheochromocytoma or acute central nervous system injury, which demonstrate direct links between catecholamine surges and the syndrome [18,19,20]. Additionally, recent studies have highlighted the influence of brain–heart interactions, particularly involving limbic network dysfunction and hypothalamic–pituitary–adrenal axis hyperactivity [6,21]. The observation that microRNAs (miR) involved in TS share expression profiles with those implicated in major depression suggests that epigenetic factors also contribute to its pathogenesis [22].

Given the complex interplay between neurohormonal, genetic, and environmental factors, it is unsurprising that the search for definitive genetic risk factors has yielded inconclusive results [23]. Nonetheless, the identification of familial cases with identification of a large array of genes involved [24,25,26,27,28,29,30], and female sex prevalence supports the hypothesis of a polygenic background. In this review, we examine the most relevant genetic variants implicated in TS and explore future directions for identifying a multigene risk panel to guide clinical diagnosis and risk stratification.

## 2. Methodology

This review was conducted in accordance with the PRISMA (Preferred Reporting Items for Systematic Reviews and Meta-Analyses) guidelines [31]. A comprehensive literature search was performed using the PubMed, Scopus, and Google Scholar databases, covering publications from 1 January 2004 to 10 February 2025. The search was restricted to articles written in English.

Search terms included combinations of the following keywords: “gene”, “genetic”, “genetics”, “genome”, “SNP”, and “gene polymorphism(s)” with “Takotsubo”, “Tako-tsubo”, “Apical ballooning syndrome”, “cardiomyopathy”, “happy heart syndrome”, “broken heart syndrome”, “stress cardiomyopathy”, and “acute heart failure”.

**Figure 1:**
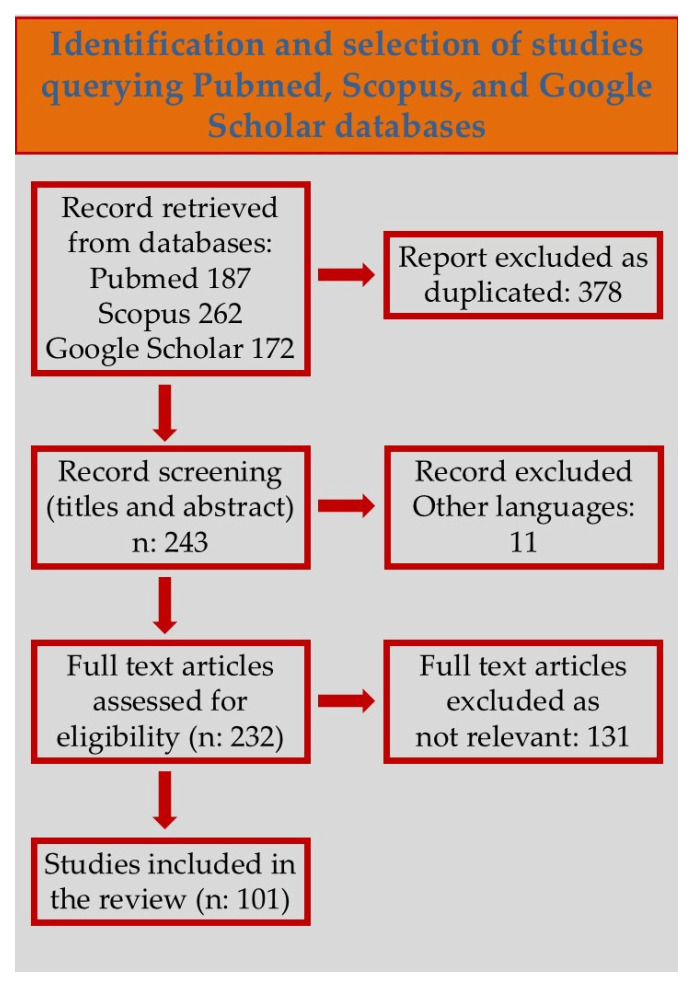
Article selection flowchart according to PRISMA statements.

Following the initial search, 621 papers were identified. After duplicate removal, language restriction, and relevance screening (as shown in Figure 1), a total of 131 articles were selected for full-text analysis. These were chosen based on their scientific quality and relevance to the genetic and pathophysiological aspects of Takotsubo syndrome.

The selected studies include original research articles, reviews, case reports, and genome-wide association studies (GWASs) that provide insight into the genetic background and mechanisms involved in TS susceptibility and pathogenesis.

This methodological approach aimed to ensure a comprehensive and up-to-date overview of the current state of knowledge regarding the genetic landscape of stress-induced cardiomyopathy.

## 3. Result and Discussion

Initial investigations into the genetic basis of TS focused on the search of candidate genes. As is known, this approach is based on the study of variants of genes that code for molecules implied in the pathogenic mechanisms of a given disease. Therefore, the analyses were focused on the polymorphisms of gene coding for the adrenergic receptors and/or in their signaling pathway transduction and regulation.

### 3.1. The Genetic Approach to Adrenergic Receptor Pathways

Early studies failed to find a significant association between TS and common polymorphisms in alpha or beta adrenergic receptors [32]. Handy et al. [33] examined key variants in *ADRB1* (rs1801252, rs1801253) and *ADRA2C* (rs61767072) in a familial case of TS, finding homozygosity for wild-type alleles and no rare mutations. In contrast, Vriz et al. [34], analyzing beta1 and/or beta2 adrenergic receptor polymorphisms in a group of 61 TS patients, reported an increased frequency of the minor allele homozygote for *ADRB1* rs1801253 and *ADRB2* rs1042714. Their subsequent 2015 study reinforced this finding, suggesting that ADRβ1rs1801253, ADRβ2 rs1042713, and rs1042714 SNPs might be risk factors for TS rather than for STEMI (ST-elevation myocardial infarction) patients [15]. However, other studies do not confirm these results [35,36].

Attention has also been directed toward G-protein-coupled receptor kinases (GRKs), particularly GRK2 and GRK5, which regulate β-adrenergic receptor signaling through receptor phosphorylation and desensitization. GRK2 is upregulated during acute TS and may play a protective role by limiting excessive adrenergic signaling [37,38]. Spinelli et al. [39] found an increased frequency of the T allele of the *GRK5* rs17098707 polymorphism in TS patients. Both in isolated cells and in transgenic mice, it has been demonstrated that the *GRK5* rs17098707 variant causes a negative inotropic effect under conditions of acute catecholamine stimulation due to βAR desensitization, inducing an imbalance between α1 coronary vasoconstriction and βAR vasodilation [39]. In spite of this functional evidence, subsequent studies, including those by our group [35], yielded inconsistent results, and the role of GRK5 variants in TS remains inconclusive [6,36,40]. Altogether, these findings suggest that the adrenergic signaling pathway should be analyzed as a whole and that variants of other genes regulating catecholamine signal transduction could play a role in susceptibility to TS.

In this respect, a different approach, as an extended exon sequencing of genes involved in adrenergic signaling pathways, gives some interesting results. Data by Goodloe et al. [41] obtained in a small TS group (28 subjects) support genetic heterogeneity in TS susceptibility and a likely polygenic basis. Beyond candidate gene studies, this research group [41] conducted an extended exon sequencing of 55 genes involved in adrenergic signaling. Their findings supported the notion of genetic heterogeneity and a polygenic basis for TS. Notably, several rare variants were shared among different TS patients, including variants in *ADH5*, *EPHA4*, *CACNG1*, and *PRKCA*—genes with critical roles in adrenergic signaling and cardiac function:

• *ADH5* encodes S-nitrosoglutathione reductase (GSNOR), a modulator of vascular tone and contractility. A missense mutation (Val346Glu) was associated with impaired responses to adrenergic stimulation [41]. Actually, in experimental models Adh5-deficiency provokes reduced inotropy and persistent systemic vasodilation in responses to beta-adrenergic stimulation [42].
• *EPHA4*, encoding a tyrosine receptor implicated in vascular tone and norepinephrine production regulation [43], harbored a deleterious G180W substitution [41].
• *CACNG1*, a subunit of the L-type calcium channel, was affected by a substitution (L123F) [41] that may impair calcium influx regulation [44].
• *PRKCA*, encoding protein kinase C alpha, regulates cardiomyocyte contractility by directly targeting protein phosphatase inhibitor-1, G protein-coupled receptor ki-nase (GRK)-2, myofilament components, and L-type calcium channels [45]. The V344L variant may affect calcium handling and contribute to contractile dysfunction [41].

These findings suggest that rare variants in genes regulating β-adrenergic signaling may synergistically increase vulnerability to catecholamine-induced myocardial injury, thus predisposing individuals to TS. In addition, six of the TS cases studied by Goodloe et al. [41] show variants affecting calcium channel subunit functionalities.

**Take to home Message:** It is possible that the presence of common and rare variants of the β-adrenergic signaling may act synergistically with other signaling pathways, increasing myocardial vulnerability and risk for TS.

### 3.2. Sex-Linked Genetics Differences in TS Susceptibility

The striking female predominance in TS—particularly among postmenopausal women—has prompted substantial investigation into sex-linked factors influencing disease susceptibility. One illustrative case involved a woman with TS who harbored a mutation in the *FMR1* gene, which is associated with fragile X syndrome and often leads to premature ovarian failure and premature menopause—a known predisposing factor for TS [46]. In addition, the protective effects of estrogen on oxidative stress, inflammation, and adrenergic signaling modulation assigns to these hormones a pivotal role in regulating interactions between different metabolic pathways that may impinge on cardiac susceptibility to TS.

Sex hormones, particularly estrogens, exert well-documented cardioprotective effects. Estrogen deficiency, as occurs in menopause, contributes to endothelial dysfunction, impaired vasodilation, and increased sympathetic tone [47]. Estrogens enhance nitric oxide (NO) synthesis by upregulating endothelial nitric oxide synthase (eNOS), inhibiting vascular apoptosis, and modulating the renin–angiotensin system via upregulation of angiotensin II type 2 receptors [48]. Estrogens also influence lipid metabolism by lowering LDL and raising HDL cholesterol levels.

Experimental animal models further support the protective role of estrogens. In ovariectomized rats subjected to emotional stress, estrogen treatment attenuated cardiac dysfunction and downregulated neuronal activation markers (e.g., c-Fos) in the brain and heart [49,50]. Estrogen supplementation also upregulated the expression of cardioprotective molecules such as atrial natriuretic peptide (ANP) and heat shock protein 70 (HSP70).

Paradoxically, elevated estradiol levels have been observed during acute TS episodes [51]. It has been hypothesized that stress-induced peripheral aromatization of androgens may lead to increased estrogen levels, possibly acting as a trigger once a critical threshold is reached [52]. Estrogens are also known to modulate the activity of GSNOR, which, as above reported, is a key regulator in adrenergic signaling [41].

Recent studies have highlighted the role of the G-protein-coupled estrogen receptor (GPER), which mediates rapid, non-genomic signaling pathways. In animal and in vitro models, GPER activation prevented adverse cardiac remodeling and reduced markers of heart failure (e.g., BNP, lactate), in part by modulating β2-adrenergic receptor internalization and Gαi signaling [53,54,55].

Additionally, a novel link between estrogen signaling and iron metabolism has emerged. Wang et al. [3] demonstrated that estrogen-mediated cardioprotection involves SmgGDS, a GTP-exchange factor that protects against ferritinophagy-induced ferroptosis—a form of iron-dependent programmed cell death implicated in myocardial injury [56,57]. SmgGDS activates AMPK/mTOR pathways, and its knockdown abolishes the protective effects of estradiol.

Finally, estrogen levels might play a role in sex-based differences in immune response that may also contribute to TS risk. Women typically exhibit stronger pro-inflammatory responses and higher expression of inflammatory markers, which may predispose them to TS and related cardiac dysfunction [58,59].

Altogether, these data support a multifaceted model in which sex hormones, immune response genes, and sex-linked polymorphisms interact to shape the markedly higher susceptibility to TS observed in women.

In spite of the evidences indicating that genetic polymorphisms in estrogen receptor genes and in their transduction pathway might be key components of the genetic susceptibility to TS, very few studies have been performed. A case-control study (81 women enrolled: 22 with TS, 22 with acute myocardial infarction, and 37 asymptomatic healthy controls) found that postmenopausal women carrying the T allele at *ESR1* rs2234693 and/or *ESR2* rs1271572 had an increased risk of developing TS [60]. Similar results were obtained in a smaller TS patient group [61].

**Take to home Message:** A systematic evaluation of the different components of the metabolic pathways regulated by estrogens appears mandatory, considering both nuclear signaling pathway and transduction mechanisms of estrogen receptors such as the emerging role of GPER.

### 3.3. Inflammation and Oxidative Stress Genes

Inflammation is one of the major players in TS pathogenesis [16]. Catecholamine excess, a key pathogenic feature, may induce endothelial injury, leading to the release of growth factors (e.g., VEGF, EGF) and cytokines that trigger inflammatory cascades. Elevated serum levels of interleukins IL-6 and IL-10 have been associated with adverse prognosis and higher in-hospital complication rates in TS patients [16,62].

Experimental models support these observations. Activation of β-adrenergic receptors in mouse cardiomyocytes induces the NLRP3 inflammasome, leading to IL-18 release, galectin-3-mediated macrophage infiltration, and pathological cardiac remodeling [63,64]. Pharmacologic inhibition of galectin-3 or genetic deletion of NLRP3/IL-18 attenuates this inflammatory damage, underscoring the relevance of this pathway.

Analyses of heart tissue during acute TS have shown an increase in both resident and infiltrating monocyte/macrophage populations [12,65]. There was no difference in the total circulating monocyte count during the acute phase, but myocardial tissue analyses from TS patients reveal that monocyte/macrophage infiltration is characterized by the predominance of the non-classical CD14+CD16+ subset. This differs from acute myocardial infarction, where CD14+CD16− monocytes are more prevalent in the infarcted tissue [65]. The pathogenic relevance of this shift remains under investigation but suggests a distinct monocyte recruitment pattern in TS.

Resident cardiac macrophages—derived from yolk sac precursors—appear to exert protective, anti-inflammatory roles, whereas infiltrating macrophages from circulating monocytes promote inflammation and injury [66,67].

Macrophages express on their surface a large set of receptor molecules able to recognize both specific antigens and conservative motifs on pathogens (PAMPs-pathogen associated molecular patterns) through Toll-like receptors (TLRs), key components of innate immunity. TLRs are also implicated in the recognition of the molecular patterns of endogenous host material that is released during cellular injury-damage associated molecular patterns (DAMPs-damage associated molecular patterns). DAMPs (damage-associated molecular patterns), such as self-DNA from necrotic cardiomyocytes, activate intracellular DNA sensing pathways that lead to proinflammatory cytokine release (e.g., TNF-α, IL-6, CCL2, IFN-β) and further myocardial injury [68,69,70]. In animal models, isoproterenol administration increases TLR2, TLR4, and TLR6 expression in cardiomyocytes and infiltrating immune cells. TLR activation contributes to oxidative stress, cytokine release, and apoptosis of cardiomyocytes and endothelial cells [71,72,73]. Similar results have been obtained in human TS patients [74]. In this view, as suggested by Kołodzińska [73], genetic polymorphisms of *TLR*s genes might be involved in susceptibility to TS as demonstrated for other cardiac pathologies [75,76].

The proteomic profile of acute TS patients is characterized by increased inflammatory mediators, such as CRP, SAA2, and SAA1, and stress hormones, according to left ventricular dysfunction [77]. Accordingly, an increase of proinflammatory cytokine levels might be observed, and IL-6, IL-2, IL-4, TNF-α, INF-γ, and CXCL1 increase has been suggested as serum signature of TS acute phase [78]. In a preliminary study, our group has explored the role of inflammatory gene polymorphisms in TS susceptibility. Although single cytokine gene variants (e.g., *IL-1A*, *IL-1B*, *IL-6*, *TNF-α*, *IL-10*, *TGF-β*) did not differ significantly in TS patients compared to controls, the combination of *IL-10* rs1800872CC genotype and *GRK5* rs2230345T allele was associated with increased TS risk [79]. This suggests a potential synergistic effect between adrenergic and inflammatory pathways deleterious genetic variants.

Interestingly, rare variants in genes of the TGF-β pathway may also predispose to TS. Case reports of patients with Loeys–Dietz syndrome (LDS), a connective tissue disorder caused by mutations in TGF-β-related genes, have described TS onset. One patient carried a *TGFB2* variant, previously linked to sporadic ascending aortic aneurysms in women [80,81,82]. These findings raise the possibility that TGF-β signaling dysfunction contributes to structural or inflammatory vulnerability in TS.

Oxidative stress is another hallmark of TS pathogenesis. Catecholamine oxidation and stress-induced neuropeptides increase ROS (reactive oxygen species) production, leading to cellular injury, impaired calcium handling, energy depletion, and contractile dysfunction [83]. ROSs also impair NO production via eNOS uncoupling and promote inflammatory cytokine release, perpetuating vascular damage [84,85].

The oxidative stress-related enzyme heme oxygenase-1 (HO-1) is upregulated in cardiac macrophages following emotional stress. While HO-1 is cytoprotective in some contexts, it also promotes free iron release and ferritin upregulation, which may trigger ferroptosis, an iron-dependent form of cell death involved in post ischemic reperfusion injury, characterized by dysregulated iron metabolism and lipid peroxidation [56,83].

Using genome-wide expression analysis, Nef et al. [86] evaluated the modification of whole gene expression in a metabolic pathway and integrated a functional cluster in the heart tissue of TS patients. This research group demonstrated the upregulation of the Nrf2-mediated antioxidant response pathway, including increased expression of SOD, catalase, and GPX1, suggesting a compensatory mechanism to counter ROS injury [86].

NADPH oxidases, particularly NOX4 and p67phox, are major sources of ROS in the myocardium. In animal models, *NOX4* gene deletion or inhibition reduced ROS production, NLRP3 inflammasome activation, and cardiac dysfunction following β-adrenergic stimulation [63,87].

In this view, it appears to be of a certain relevance that the minor allele of the *NOX4* rs11018628 variant has been associated with reduced ischemic stroke risk and may influence ROS generation and cardiovascular outcomes [88,89,90]. Intriguingly, this variant has also been linked to other conditions, such as bone density loss and psoriasis, supporting its role in systemic inflammatory and oxidative processes [91].

**Take to home Message:** Data from TS related inflammation and oxidative stress evaluation highlight that takotsubo pathogenesis involves a complex interplay of inflammation and oxidative stress. Genetic variants affecting cytokine signaling, TLR function, ROS production, and antioxidant responses may contribute to individual susceptibility. These findings underscore the importance of a multigenic, systems-level approach to delineate genetic signature of TS, potentially useful in predicting TS risk.

### 3.4. Heart Brain Interaction and TS: Genetic and Epigenetic Markers of Susceptibility

Data from large registries indicate that more than 50% of TS patients have a history of psychiatric or neurologic disorders, suggesting a key role of central autonomic network dysfunction in the pathogenesis of the syndrome [59]. The heart–brain axis appears to be particularly relevant, as stress-induced activation of the sympathetic nervous system is a major trigger for TS. The mechanisms involved in central nervous system triggering of TS involves both genetic and epigenetic factors.

The coronary microcirculation, richly innervated by sympathetic fibers [92], is susceptible to acute neurogenic insults. Emotional or physical stress can activate the hypothalamic–pituitary–adrenal (HPA) axis, leading to excessive catecholamine release and subsequent myocardial dysfunction in predisposed individuals. Conditions such as pheochromocytoma, thyrotoxicosis, and subarachnoid hemorrhage—associated with catecholamine storms—are well-known precipitants of TS [6,67]. Most frequently, TS affects post-menopausal women with pre-existing psychiatric or neurological illnesses or with a history of substance abuse [93].

Another mechanism of the interaction between CNS and cardio circulatory system that might be implied in TS surge is the hypothalamic–pituitary–adrenal axis that is highly sensitive to emotional stress. A minority of pheochromocytoma patients have TS symptoms and clinical signs [18]. A recent study reports that 10% of pheochromocytoma female patients had confirmed takotsubo syndrome, whereas none were found in the males, confirming the sex-linked differences in susceptibility observed in the generality of TS patients [19]. One study investigating adrenergic receptor polymorphisms in patients with pheochromocytoma or paraganglioma found that carriers of the *ADRA2C* Del322–325 variant had increased susceptibility to catecholamine-induced cardiomyopathy, including TS-like presentations [20]. This supports the notion that variants in genes regulating extracardiac adrenergic tone modulate individual vulnerability to stress-induced cardiac dysfunction.

Epigenetic factors may also bridge the neuropsychiatric and cardiac aspects of TS. MicroRNAs (miRs) involved in emotional regulation and depression, particularly miR-16 and miR-26a, are upregulated in TS patients and distinguish them from those with STEMI or healthy controls [22]. These miRs modulate G-protein signaling pathways in cardiomyocytes by targeting GNB1 and RGS4, key components of β-adrenergic signal transduction [94,95,96].

Couch et al. demonstrated that overexpression of miR-16 and miR-26a in cardiomyocytes alters the apical response to adrenaline, mimicking the regional contractility abnormalities seen in TS. Their synergistic action reduces apical inotropy by impairing β-adrenergic receptor signaling through Gαs-Gαi switch modulation [94].

Neurocardiac miR profiles observed in TS partially overlap with those seen in major depressive disorder, highlighting shared molecular pathways between emotional dysregulation and stress cardiomyopathy [22].

There are also evidences that miRs are implicated in genetic susceptibility to TS even at the cardiac tissue level [97,98,99,100]. In particular, d’Avenia et al. [98] described a posttranscriptional regulation pathway leading to Bcl2-associated athanogene 3 (*BAG3*) induction on epinephrine stimulation. Activation of BAG3 expression after epinephrine stimulation is mediated by a miRNA (miR-371a-5p) that binds the 3′-untranslated region (3′-UTR) increasing BAG3 transcription. Genotyping of 70 TS patients allowed us to identify a particularly frequent mutation in the 3′-UTR (SNP rs8946) of *BAG3* [98] that abolishes the miR-371a-5p (miR-371a) responding motif. Considering that BAG3 induction appears to be a required component for correct sarcomere assembly in cardiomyocytes, the rs8946 variant may potentially represent a new genetic risk marker for TS.

**Take to home Message:** Evidence suggests that neuropsychiatric comorbidities in TS are not merely epiphenomena but may reflect underlying genetic and epigenetic vulnerabilities. These findings reinforce the importance of integrating neuropsychiatric screening and autonomic nervous system assessment in patients at risk for TS, while also pointing to novel avenues for biomarker discovery and personalized risk stratification.

### 3.5. The Genetic Approach to (Micro) Vascular and Endothelial Damage Hypothesis

One of the suggested mechanisms of acute insurgent TS is the possibility that a rupture of atherosclerotic plaque might trigger a transient thrombotic coronary occlusion followed by a fast-dissolving clot [101]. However, anatomically, the region of myocardium with morphological modification in TS is more extended than the perfusion area of a single epicardial coronary artery. Moreover, the results of instrumental investigations, even if conducted with the most advanced methodologies, did not show occlusion pictures during acute TS [101,102]. In spite of the lack of demonstration of the presence of a transient intracoronary thrombotic occlusion, the frequency of the most common prothrombotic genetic variants has been studied in TS patients [103,104,105]. Even if a clear demonstration of thrombus formation in the coronary circulation is lacking, a reduced vascular flow and presence of endothelial cell necrosis in myocardial biopsies of TS patients and in animal models suggest that damages of microvascular coronary circulation could play a relevant role in TS pathophysiology [106].

Actually, endothelial dysfunction, due to an imbalance of vasoconstriction- and vasodilation-associated signaling (e.g., imbalance between vasoconstrictor signals endothelin-1 (ET-1) and vasodilator molecules as nitric oxide (NO)) might be one of the central mechanisms for development of cardiovascular diseases [107].

Recently, two papers have focused the attention on the role that ionic channels on endothelial cells from microvascular vessels and smooth muscle cells might play in TS pathogenesis. In an experimental animal model characterized by a genetically determined lack of potassium voltage-gated channel, shaker-related subfamily, member 5, also known as KCNA5 or Kv1.5 channel, has demonstrated that smooth muscle cells of coronary arterioles of Kv1.5−/− mice submitted to vascular stress showed a downregulation of genes involved in pathways of oxidative phosphorylation and an upregulation of genes involved in pathways of hypoxia [13]. This upregulation occurred in the base and not in the apex of left ventricle, even if the apex has reduced perfusion compared to the base [13].

Using isolated endothelial cells from microvascular vessels (HCMECs), Yang et al. [108] analyzed the expression of genes coding for Adrenoceptors, alpha-1 (ADRA1A), alpha-2 (ADRA2A), ion channels (calcium-activated potassium channel type 1–3 and 4: KCNN1, KCNN2, KCNN3, KCNN4), big conductance calcium-activated potassium channel (KCNMA1), endothelin-1 (EDN1), and NADPH oxidases (NOX1, NOX2 and NOX5).

Data obtained in this in vitro model indicated that high dose epinephrine or phenylephrine stimulation of adrenoceptors can activate KCNN1-3 channels leading to the increase of ET-1 and ROS production; furthermore, activation of α1-adrenoceptors increase ROS and ET-1 generation also in KCNN1–3 independent ways. It is of a certain relevance that a full exome sequencing study [109], performed on seven female unrelated TS patients, evidenced that four of the seven patients were carriers of a non-synonymous coding change (rs1805124) in *SCN5A* gene, which codes for voltage-gated ion channels. The presence of mutated *SCN5A* gene was found associated to different types of cardiomyopathies (familial atrial and ventricular fibrillation, different types of heart block, Brugada Syndrome, dilated cardiomyopathy, and sick sinus syndrome) [110]. Similar results were obtained in a subsequent study on a patient with a coexistence of Brugada syndrome and takotsubo cardiomyopathy [111].

**Take to home Message:** A systematic evaluation of the role genetic variability of different types of ion channels in TS would potentially add new pieces of evidence to the puzzle that characterizes the TS genetic background.

### 3.6. Genome-Wide Association Studies (GWASs) on TS

Genome-wide association studies (GWASs) represent a powerful tool for uncovering genetic variants associated with complex diseases, including Takotsubo syndrome (TS). Unlike candidate gene approaches, GWASs scan the entire genome to identify statistically significant associations between single nucleotide polymorphisms (SNPs) and disease traits, irrespective of prior biological assumptions.

Eitel et al. conducted the first GWAS on TS, analyzing a relatively small cohort of 96 patients and 475 healthy controls. Despite the limited sample size, the study identified 18 SNPs with strong linkage disequilibrium that were associated with traits relevant to TS (OR range 0.32 to 3.19; *p* statistical significance 1.30 × 10^−5^ to 5.24 × 10^−7^), including psychiatric disorders, heart rate variability, blood pressure, thyroid hormone regulation, and lipid metabolism [112]. These findings support the hypothesis that TS arises from a complex interplay of genetic predispositions across multiple biological systems. The 18 SNPs identified by Eitel et al. [112] lie both on functionally characterized DNA sequences and on regions that are not yet well characterized. Eight SNPs (rs1154275, rs12612435, rs13179382, rs17146144, rs4605019, rs4812257, rs62253104, rs72970558) tagged functionally uncharacterized DNA sequences. We queried the NCBI dbSNP database (http://www.ensembl.org/index.html, accessed on 7 March 2025) to discuss whether the functionally identified DNA sequence products tagged by the relative SNP might play a role in the predisposition or pathogenesis of TS (Table 1).

Among the SNPs identified, several were located in or near genes with plausible functional relevance:

• *GRM7* (rs113154180): Encodes the metabotropic glutamate receptor 7, previously implicated in mood disorders and autism spectrum conditions, suggesting a neuropsychiatric link [113].
• *RBFOX1* (rs12444925): Involved in RNA splicing and regulation of cytoskeletal genes in cardiomyocytes; downregulation is associated with heart failure progression [114].
• *PIWIL2* (rs13273616): Encodes a piRNA-associated protein involved in endothelial cell dysfunction under hypoxic stress, potentially linking oxidative stress to TS pathogenesis [115,116].
• *SLC5A7* (rs4676168): Codes for a high-affinity choline transporter essential for acetylcholine synthesis, reflecting parasympathetic modulation of cardiac tone that might be paradoxically modified in TS patient after acetylcholine infusion [117,118].
• *CNGB3* (rs4961212): Although primarily involved in visual transduction [119], its homology to cardiac ion channels raises the possibility of shared regulatory mechanisms affecting excitability and calcium handling [120].
• SEMA3D (rs6944978): Encodes semaphorin 3D, a guidance molecule essential for coronary artery development and sympathetic innervation of the heart [121,122,123].
• *ADAMTS5* (rs162487) and *ADAM10* (rs56403110): Both metalloproteinases are involved in extracellular matrix remodeling and inflammation, processes that contribute to cardiac remodeling and injury [124,125,126,127,128].
• Some variants were located in non-coding RNAs or regions with regulating functions:
• *LINC02625* (rs7070797): A long intergenic non-coding RNA potentially interacting with apoptotic regulators [129].
• *LY86-AS1* (rs9392780): Antisense RNA downregulated in cerebral hemorrhage and diabetes, implicating it in vascular or metabolic comorbidities [130,131].

Additionally, recent GWAS data presented by Hakansson and Tornvall at the ESC Congress 2024 identified *BTN2A2* (rs1614887) as a novel candidate gene. *BTN2A2* encodes a glycoprotein receptor involved in lipid metabolism and is highly expressed in cardiac tissue [132]. Variants in this gene family have also been linked to major depressive disorder, reinforcing the bridge between psychiatric vulnerability and TS [133].

Some of the newly identified TS-associated SNPs overlap with those found in inflammatory skin diseases such as psoriasis, which is known to increase cardiovascular risk. For example, genes such as *SOCS3*, *BCL3*, *STAT5A*, and *OSM*—all regulated by IL-6 and the JAK/STAT pathway—may play dual roles in inflammation and vascular remodeling [134].

**Take to home Message:** GWAS findings, while still preliminary, underscore the polygenic nature of TS and highlight the convergence of multiple biological pathways—neurological, inflammatory, hormonal, metabolic, and structural—in shaping disease risk. Larger, multicentric GWAS efforts with robust clinical phenotyping are needed to validate these findings and construct a predictive genetic risk model for TS.

## 4. Conclusions and Future Perspectives

Takotsubo syndrome (TS) is a complex, multifactorial condition resulting from the dynamic interplay of environmental triggers, neurohormonal imbalances, and genetic susceptibility. While the pathophysiological mechanisms have been increasingly elucidated—particularly the role of catecholaminergic overstimulation—the genetic underpinnings of TS remain largely not well understood.

A growing body of evidence suggests that TS does not follow a monogenic inheritance model but is instead influenced by a polygenic architecture, where multiple rare and common variants contribute to individual vulnerability.

Genome-wide approaches have opened new avenues for exploring the genetic landscape of TS, uncovering novel associations with genes not previously linked to the syndrome, including those involved in psychiatric disorders, lipid metabolism, and cardiac structural integrity. However, current GWASs are limited by small sample sizes and phenotypic heterogeneity.

**Figure 2:**
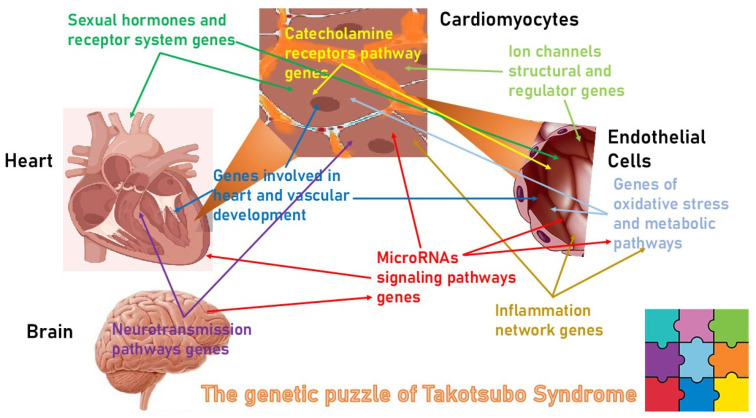
The genetic puzzle of Takotsubo cardiomyopathy. Genetic susceptibility to Takotsubo cardiomyopathy may be the result of a complex interaction among different functional and metabolic pathways that can be modified by the presence of common or rare variants reciprocally interacting that increase the sensitivity of cardiomyocyte and microvascular coronary to adrenergic overstimulation or susceptibility to inflammatory oxidative and/or apoptotic damage.

Taken together, these data allow us to define TS as a genetic puzzle in which variations in different sets of genes and metabolic pathways in individual patients can favor the onset of the disease by modifying from time to time the sensitivity to adrenergic overstimulation or susceptibility to inflammatory oxidative or apoptotic damage or structural defects of contractile apparatus or signal transmission (Figure 2).

What emerges from the enormous amount of data that have been published so far is that, especially for studies conducted with genome scanning methodologies, identification of gene associated to TS risk is a common feature. These genetic variations affect many functional and metabolic pathways crucial for TS pathogenesis. At the same time, the continuous evolution of the classification and diagnosis criteria of the pathology has made evident the breadth of the spectrum of clinical TS phenotypes. These two factors, on the one hand have so far prevented a clear definition of the genetic risk profile for TS; on the other, they indicate the direction in which efforts of the future studies should be directed.

Future research should focus on large-scale, multicenter studies integrating high-resolution genomic techniques (e.g., whole-exome sequencing, transcriptomics, epigenomics) with detailed clinical phenotyping. Particular attention should be given to sex-specific genetic effects, neurocardiac interactions, and the identification of endophenotypes within the broader TS spectrum.

The development of multigene susceptibility panels, potentially enriched by machine learning and network-based analysis, could pave the way for improved risk stratification, early identification of at-risk individuals, and targeted prevention strategies. Furthermore, a better understanding of the genetic basis of TS may facilitate the discovery of novel therapeutic targets aimed at modulating adrenergic signaling, inflammation, and myocardial resilience to stress.

In conclusion, the genetic architecture of Takotsubo syndrome is gradually emerging. An integrative systems investigation, combining advances in genomics, transcriptomics, and data-driven modeling, offers promising avenues to decode its complexity and transform molecular discoveries into precision medicine strategies for this still enigmatic and fascinating pathology.

## Figures and Tables

**Table 1:** Functionally and not functionally characterized DNA sequences tagged by the SNPs identified in GWAS of stress induced cardiomyopathy subjects by Eitel et al. [112].

SNP	Chromosome (GRCh38)	Characterized DNA Sequences	Variation Type	Functional Role
rs113154180	3:7563703	*GRM7*	Intron variant	Major depression susceptibility
rs12444925	16:5420155	*RBFOX1*	Genic downstream transcript variant, intron variant	Cytoskeletal organization, in cardiomyoblast
rs13273616	8:22333990	*PIWIL2*	Intron variant, Genic downstream transcript variant,	Interaction with JAK2/STAT3 in endothelial cells in hypoxic condition
rs162487	21:26940919	*ADAMTS5*	Intron variant	Angiogenesis and inflammation
rs4676168	2:107987046	*SLC5A7*	Genic downstream transcript variant, intron variant	Cholin trasporter in the acetylcholine neuro-transmission pathway
rs4961212	8:86709209	*CNGB3*	Genic downstream transcript variant, intron variant	Achromatopsia, cyclic nucleotide-gated (CNG) channels
rs56403110	15:58718163	*ADAM10*	Intron variant	Molecular scissors for extracellular domains Reduction in oxidative stress, Anti apoptosis
rs6944978	7:85184930	*SEMA3D*	Genic downstream transcript variant, intron variant	Cardiovascular development
rs7070797	10:61792015	*LINC02625*	Intron variant	miR interaction, Apoptosis
rs9392780	6:6405398	*LY86-AS1*	Intron variant	Hemorrhagic Ictus, IDDM

*GRM7*: glutamate metabotropic receptor 7; *RBFOX1*: RNA binding fox-1 homolog 1; *PIWIL2*: piwi like RNA-mediated gene silencing 2; *ADAMTS5*: ADAM Metallopeptidase with Thrombospondin Type 1 Motif 5; *SLC5A7*: solute carrier family 5 member 7, *CNGB3*: cyclic nucleotide-gated ion channel; *ADAM10*: ADAM Metallopeptidase Domain 10; *SEMA3D*: Semaphorin 3D; *LINC02625*: Long Intergenic Non-Protein Coding RNA 2625; *LY86-AS1*: LY86 antisense RNA 1.

## Data Availability

Being a review, no new data were created and analyzed in this study.

## Abbreviations

The following abbreviations are used in this manuscript:

ADAM10	Adam Metallopeptidase Domain 10
ADAMTS5	Adam Metallopeptidase with Thrombospondin Type 1 Motif 5
ADH5	Alcohol Dehydrogenase 5
ADRA	Adrenergic Receptor Alpha
ADRB	Adrenergic Receptor Beta
AMI	Acute Myocardial Infarction
AMPK	Adenosin Monophosphate Kinase
ANP	Atrial Natriuretic Peptide
AR	Adrenergic Receptor
BAG3	BCL2-Associated Athanogene 3
BLC3	B-cell lymphoma 3
BNP	Brain Natriuretic Peptide
BTN2A2	Butyrophilin Subfamily 2 Member A2
CACNG1	Calcium Channel Gamma-1
cAMP	Cyclic Adenosine 3,5-Monophosphate
CCL2	CC Motif Chemokine 2
CCL20	CC Motif Chemokine 20
CCL5	CC Motif Chemokine 5
CD	Cluster Of Differentiation
CNGB3	Cyclic Nucleotide-Gated Ion Channel
CNS	Central Nervous System
CRP	C-Reactive Protein
CXCL1	CXC Motif Chemokine 1
DAMPs	Damage Associated Molecular Patterns
EDN1	Endothelin-1
EGF	Endothelial Growth Factor
eNOS	Endothelial Nitric Oxide Synthase
EPHA4	Ephrin Type-A Receptor 4
ESC	European Society of Cardiology
ESR1 -2	Estrogen Receptor-1 -2
ET-1	Endothelin-1
FMR1	Fragile X Messenger Ribonucleoprotein 1
GNB1	Guanine Nucleotide-Binding Protein Beta-1
GPER	G Protein-Coupled Estrogen Receptor
GRK	G Protein Coupled Receptor Kinase
GRM7	Glutamate Metabotropic Receptor 7
GSNOR	S-Nitrosoglutathione Reductase
GWAS	Genome-Wide Association Studies
Gαi	Inhibitory G-Protein
Gαs	G-Protein-Activated
HCMECs	Human Endothelial Cells from Microvascular Vessels
HDL	High density lipoprotein
HO-1	Heme Oxygenase-1
HPA	hypothalamic–pituitary–adrenal
IFN	Interferon
IL	Interleukin
KCNA5	Potassium Voltage-Gated Channel, Member 5
KCNMA1	Big Conductance Calcium-Activated Potassium Channel
KCNN1-4	Calcium-Activated Potassium Channel Type 1–4
Kv1.5	Potassium Voltage-Gated Channel
LDL	Low density Lipoprotein
LDS	Loeys-Dietz Syndrome
LINC02625	Long Intergenic Non-Protein Coding RNA 2625
LY86-AS1	LY86 Antisense RNA 1
miR	MicroRNA
mTOR	Mechanistic Target of Rapamycin
NADP	n-adenil Diphosphate
NLRP3	NOD-Like Receptor Pyrin Domain-Containing 3
NO	Nitric Oxide
NOX	NADPH Oxidase
Nrf2	Nuclear Factor [Erythroid-Derived 2]-Like 2
OSM	Oncostatin M
PAMPs	Pathogen Associated Molecular Patterns)
PIWIL2	Piwi Like RNA-Mediated Gene Silencing 2
PRISMA	Preferred Reporting Items for Systematic Reviews and Meta-Analyses
PRKCA	Protein Kinase C, Alpha
RBFOX1	RNA Binding Fox-1 Homolog 1
RGS	G Protein-Signaling
ROS	Reactive Oxygen Species
rs	Reference SNP
SAA-1 -2	Serum Amyloid A-1 -2
SCN5A	Sodium Channel Protein Type 5 Subunit Alpha
SEMA3D	Semaphorin 3D
SLC5A7	Solute Carrier Family 5 Member 7
SmgGDS	GTP-Binding Protein GDP Dissociation Stimulator
SNP	Single Nucleotide Polymorphism
SOCS	Suppressor of cytokine signaling
SOD	Superoxide Dismutase
STAT5A	Signal Transducer and Activator of Transcription 5A
STEMI	ST-Elevation Myocardial Infarction
TGF-β	Transforming Growth Factor-β
TLR	Toll-Like Receptor
TNF	Tumor Necrosis Factor
TS	Takotsubo Syndrome
VEFG	Vascular Endothelial Growth Factor

## References

[1] Abe Y., Kondo M. (2003). Apical ballooning of the left ventricle: A distinct entity?. Heart.

[2] Shufelt C.L., Pacheco C., Tweet M.S., Miller V.M. (2018). Sex-Specific Physiology and Cardiovascular Disease. Adv. Exp. Med. Biol..

[3] Wang T., Xiong T., Yang Y., Chen X., Ma Z., Zuo B., Ning D., Zhou B., Song R., Liu X. (2023). Estradiol-mediated small GTP-binding protein GDP dissociation stimulator induction contributes to sex differences in resilience to ferroptosis in takotsubo syndrome. Redox Biol..

[4] Dias A., Gil I.J.N., Santoro F., Madias J.E., Pelliccia F., Brunetti N.D., Salmoirago-Blotcher E., Sharkey S.W., Eitel I., Akashi Y.J. (2019). Takotsubo syndrome: State-of-the-art review by an expert panel—Part 1. Cardiovasc. Revasc. Med..

[5] El-Battrawy I., Santoro F., Núñez-Gil I.J., Pätz T., Arcari L., Abumayyaleh M., Guerra F., Novo G., Musumeci B., Cacciotti L. (2024). Age-Related Differences in Takotsubo Syndrome: Results from the Multicenter GEIST Registry. J. Am. Heart Assoc..

[6] Moscatelli S., Montecucco F., Carbone F., Valbusa A., Massobrio L., Porto I., Brunelli C., Rosa G.M. (2019). An Emerging Cardiovascular Disease: Takotsubo Syndrome. BioMed Res. Int..

[7] Omerovic E., Citro R., Bossone E., Redfors B., Backs J., Bruns B., Ciccarelli M., Couch L.S., Dawson D., Grassi G. (2022). Pathophysiology of Takotsubo syndrome—A joint scientific statement from the Heart Failure Association Takotsubo Syndrome Study Group and Myocardial Function Working Group of the European Society of Cardiology—Part 1: Overview and the central role for catecholamines and sympathetic nervous system. Eur. J. Heart Fail..

[8] Omerovic E., Citro R., Bossone E., Redfors B., Backs J., Bruns B., Ciccarelli M., Couch L.S., Dawson D., Grassi G. (2022). Pathophysiology of Takotsubo syndrome—A joint scientific statement from the Heart Failure Association Takotsubo Syndrome Study Group and Myocardial Function Working Group of the European Society of Cardiology—Part 2: Vascular pathophysiology, gender and sex hormones, genetics, chronic cardiovascular problems and clinical implications. Eur. J. Heart Fail..

[9] Redfors B., Shao Y., Lyon A.R., Omerovic E. (2014). Diagnostic criteria for takotsubo syndrome: A call for consensus. Int. J. Cardiol..

[10] Ghadri J.R., Cammann V.L., Jurisic S., Seifert B., Napp L.C., Diekmann J., Bataiosu D.R., D’Ascenzo F., Ding K.J., Sarcon A. (2017). A novel clinical score (InterTAK Diagnostic Score) to differentiate takotsubo syndrome from acute coronary syndrome: Results from the International Takotsubo Registry. Eur. J. Heart Fail..

[11] Akhtar M.M., Cammann V.L., Templin C., Ghadri J.R., Lüscher T.F. (2023). Takotsubo syndrome: Getting closer to its causes. Cardiovasc. Res..

[12] Scally C., Abbas H., Ahearn T., Srinivasan J., Mezincescu A., Rudd A., Spath N., Yucel-Finn A., Yuecel R., Oldroyd K. (2019). Myocardial and Systemic Inflammation in Acute Stress-Induced (Takotsubo) Cardiomyopathy. Circulation.

[13] Dong F., Yin L., Sisakian H., Hakobyan T., Jeong L.S., Joshi H., Hoff E., Chandler S., Srivastava G., Jabir A.R. (2023). Takotsubo syndrome is a coronary microvascular disease: Experimental evidence. Eur. Heart J..

[14] Rosenbaum D.M., Rasmussen S.G., Kobilka B.K. (2009). The structure and function of G-protein-coupled receptors. Nature.

[15] Vriz O., Minisini R., Zito C., Boccato E., Fimiani F., Pirisi M., Facciolo C., Limongelli G., Bossone E., Calabrò P. (2015). Can apical ballooning cardiomyopathy and anterior STEMI be differentiated based on β1 and β2-adrenergic receptors polymorphisms?. Int. J. Cardiol..

[16] Dias A., Gil I.J.N., Santoro F., Madias J.E., Pelliccia F., Brunetti N.D., Salmoirago-Blotcher E., Sharkey S.W., Eitel I., Akashi Y.J. (2019). Takotsubo syndrome: State-of-the-art review by an expert panel—Part 2. Cardiovasc. Revasc. Med..

[17] Y-Hassan S. (2019). Plasma Epinephrine Level and its Causal Link to Takotsubo Syndrome Revisited: Critical Review with a Diverse Conclusion. Cardiovasc. Revasc. Med..

[18] Diaz B., Elkbuli A., Ehrhardt J.D., McKenney M., Boneva D., Hai S. (2019). Pheochromocytoma-related cardiomyopathy presenting as broken heart syndrome: Case report and literature review. Int. J. Surg. Case Rep..

[19] Ali N.A., Calissendorff J., Falhammar H. (2025). Sex differences in presentation of pheochromocytoma and paraganglioma. Front. Endocrinol..

[20] Amar J., Brunel J., Bauters C.C., Jacques V., Delmas C., Odou M.F., Savagner F. (2022). Genetic biomarkers of life-threatening pheochromocytoma-induced cardiomyopathy. Endocr. Relat. Cancer.

[21] Hiestand T., Hänggi J., Klein C., Topka M.S., Jaguszewski M., Ghadri J.R., Lüscher T.F., Jäncke L., Templin C. (2018). Takotsubo Syndrome Associated with Structural Brain Alterations of the Limbic System. J. Am. Coll. Cardiol..

[22] Bergami M., Fabin N., Cenko E., Bugiardini R., Manfrini O. (2023). MicroRNAs as Potential Biomarkers in Coronary Artery Disease. Curr. Top. Med. Chem..

[23] Ferradini V., Vacca D., Belmonte B., Mango R., Scola L., Novelli G., Balistreri C.R., Sangiuolo F. (2021). Genetic and Epigenetic Factors of Takotsubo Syndrome: A Systematic Review. Int. J. Mol. Sci..

[24] Pison L., De Vusser P., Mullens W. (2004). Apical ballooning in relatives. Heart.

[25] Cherian J., Angelis D., Filiberti A., Saperia G. (2007). Can takotsubo cardiomyopathy be familial?. Int. J. Cardiol..

[26] Kumar G., Holmes D.R., Prasad A. (2010). “Familial” apical ballooning syndrome (Takotsubo cardiomyopathy). Int. J. Cardiol..

[27] Subban V., Ramachandran S., Victor S.M., Gnanaraj A., Ajit M.S. (2012). Apical ballooning syndrome in first degree relatives. Indian Heart J..

[28] Ikutomi M., Yamasaki M., Matsusita M., Watari Y., Arashi H., Endo G., Yamaguchi J., Ohnishi S. (2014). Takotsubo cardiomyopathy in siblings. Heart Vessels.

[29] Caretta G., Robba D., Vizzardi E., Bonadei I., Raddino R., Metra M. (2015). Tako-tsubo cardiomyopathy in two sisters: A chance finding or familial predisposition?. Clin. Res. Cardiol..

[30] Ekenbäck C., Tornvall P., Spaak J. (2019). Takotsubo twins. BMJ Case Rep..

[31] Page M.J., McKenzie J.E., Bossuyt P.M., Boutron I., Hoffmann T.C., Mulrow C.D., Shamseer L., Tetzlaff J.M., Akl E.A., Brennan S.E. (2021). The PRISMA 2020 statement: An updated guideline for reporting systematic reviews. PLoS Med..

[32] Sharkey S.W., Maron B.J., Nelson P., Parpart M., Maron M.S., Bristow M.R. (2009). Adrenergic receptor polymorphisms in patients with stress (tako-tsubo) cardiomyopathy. J. Cardiol..

[33] Handy A.D., Prasad A., Olson T.M. (2009). Investigating genetic variation of adrenergic receptors in familial stress cardiomyopathy (apical ballooning syndrome). J. Cardiol..

[34] Vriz O., Minisini R., Citro R., Guerra V., Zito C., De Luca G., Pavan D., Pirisi M., Limongelli G., Bossone E. (2011). Analysis of beta1 and beta2-adrenergic receptors polymorphism in patients with apical ballooning cardiomyopathy. Acta Cardiol..

[35] Novo G., Giambanco S., Guglielmo M., Arvigo L., Sutera M.R., Giambanco F., Evola S., Vaccarino L., Bova M., Lio D. (2015). G-protein-coupled receptor kinase 5 polymorphism and Takotsubo cardiomyopathy. J. Cardiovasc. Med..

[36] Figtree G.A., Bagnall R.D., Abdulla I., Buchholz S., Galougahi K.K., Yan W., Tan T., Neil C., Horowitz J.D., Semsarian C. (2013). No association of G-protein-coupled receptor kinase 5 or β-adrenergic receptor polymorphisms with Takotsubo cardiomyopathy in a large Australian cohort. Eur. J. Heart Fail..

[37] Franco A., Sorriento D., Gambardella J., Pacelli R., Prevete N., Procaccini C., Matarese G., Trimarco B., Iaccarino G., Ciccarelli M. (2018). GRK2 moderates the acute mitochondrial damage to ionizing radiation exposure by promoting mitochondrial fission/fusion. Cell Death Discov..

[38] Nakano T., Onoue K., Nakada Y., Nakagawa H., Kumazawa T., Ueda T., Nishida T., Soeda T., Okayama S., Watanabe M. (2018). Alteration of β-Adrenoceptor Signaling in Left Ventricle of Acute Phase Takotsubo Syndrome: A Human Study. Sci. Rep..

[39] Spinelli L., Trimarco V., Di Marino S., Marino M., Iaccarino G., Trimarco B. (2010). L41Q polymorphism of the G protein coupled receptor kinase 5 is associated with left ventricular apical ballooning syndrome. Eur. J. Heart Fail..

[40] Onrat S.T., Dural İ.E., Yalım Z., Onrat E. (2021). Investigating changes in β-adrenergic gene expression (ADRB1 and ADRB2) in Takotsubo (stress) cardiomyopathy syndrome; a pilot study. Mol. Biol. Rep..

[41] Goodloe A.H., Evans J.M., Middha S., Prasad A., Olson T.M. (2014). Characterizing genetic variation of adrenergic signalling pathways in Takotsubo (stress) cardiomyopathy exomes. Eur. J. Heart Fail..

[42] Beigi F., Gonzalez D.R., Minhas K.M., Sun Q.A., Foster M.W., Khan S.A., Treuer A.V., Dulce R.A., Harrison R.W., Saraiva R.M. (2012). Dynamic denitrosylation via S-nitrosoglutathione reductase regulates cardiovascular function. Proc. Natl. Acad. Sci. USA.

[43] Zhang Z., Tremblay J., Raelson J., Sofer T., Du L., Fang Q., Argos M., Marois-Blanchet F.C., Wang Y., Yan L. (2019). EPHA4 regulates vascular smooth muscle cell contractility and is a sex-specific hypertension risk gene in individuals with type 2 diabetes. J. Hypertens..

[44] Chu P.J., Robertson H.M., Best P.M. (2001). Calcium channel gamma subunits provide insights into the evolution of this gene family. Gene.

[45] Verweij N., Benjamins J.W., Morley M.P., van de Vegte Y.J., Teumer A., Trenkwalder T., Reinhard W., Cappola T.P., van der Harst P. (2020). The Genetic Makeup of the Electrocardiogram. Cell Syst..

[46] Kleinfeldt T., Schneider H., Akin I., Kische S., Turan R.G., Nienaber C.A., Ince H. (2009). Detection of FMR1-gene in Takotsubo cardiomyopathy: A new piece in the puzzle. Int. J. Cardiol..

[47] Chou A.Y., Saw J. (2014). Basis for sex-specific expression of Takotsubo cardiomyopathy, cardiac syndrome X, and spontaneous coronary artery dissection. Can. J. Cardiol..

[48] Ghaffari S., Naderi Nabi F., Sugiyama M.G., Lee W.L. (2018). Estrogen Inhibits LDL (Low-Density Lipoprotein) Transcytosis by Human Coronary Artery Endothelial Cells via GPER (G-Protein-Coupled Estrogen Receptor) and SR-BI (Scavenger Receptor Class B Type 1). Arterioscler. Thromb. Vasc. Biol..

[49] Ueyama T., Ishikura F., Matsuda A., Asanuma T., Ueda K., Ichinose M., Kasamatsu K., Hano T., Akasaka T., Tsuruo Y. (2007). Chronic estrogen supplementation following ovariectomy improves the emotional stress-induced cardiovascular responses by indirect action on the nervous system and by direct action on the heart. Circ. J..

[50] Ueyama T., Kasamatsu K., Hano T., Tsuruo Y., Ishikura F. (2008). Catecholamines and estrogen are involved in the pathogenesis of emotional stress-induced acute heart attack. Ann. N. Y. Acad. Sci..

[51] Brenner R., Weilenmann D., Maeder M.T., Jörg L., Bluzaite I., Rickli H., De Pasquale G., Ammann P. (2012). Clinical characteristics, sex hormones, and long-term follow-up in Swiss postmenopausal women presenting with Takotsubo cardiomyopathy. Clin. Cardiol..

[52] Shansky R.M., Glavis-Bloom C., Lerman D., McRae P., Benson C., Miller K., Cosand L., Horvath T.L., Arnsten A.F. (2004). Estrogen mediates sex differences in stress-induced prefrontal cortex dysfunction. Mol. Psychiatry.

[53] Fu L., Zhang H., Machuki J.O., Zhang T., Han L., Sang L., Wu L., Zhao Z., Turley J.M., Hu X. (2021). GPER mediates estrogen cardioprotection against epinephrine-induced stress. J. Endocrinol..

[54] Prossnitz E.R., Barton M. (2011). The G-protein-coupled estrogen receptor GPER in health and disease. Nat. Rev. Endocrinol..

[55] Wang Z., Liu J., Chen Y., Tang Y., Chen T., Zhou C., Wang S., Chang R., Chen Z., Yang W. (2025). From physiology to pathology: Emerging roles of GPER in cardiovascular disease. Pharmacol. Ther..

[56] Fang X., Ardehali H., Min J., Wang F. (2023). The molecular and metabolic landscape of iron and ferroptosis in cardiovascular disease. Nat. Rev. Cardiol..

[57] Ning D., Yang X., Wang T., Jiang Q., Yu J., Wang D. (2021). Atorvastatin treatment ameliorates cardiac function and remodeling induced by isoproterenol attack through mitigation of ferroptosis. Biochem. Biophys. Res. Commun..

[58] Olivieri F., Marchegiani F., Matacchione G., Giuliani A., Ramini D., Fazioli F., Sabbatinelli J., Bonafè M. (2023). Sex/gender-related differences in inflammaging. Mech. Ageing Dev..

[59] Lam C.S.P., Arnott C., Beale A.L., Chandramouli C., Hilfiker-Kleiner D., Kaye D.M., Ky B., Santema B.T., Sliwa K., Voors A.A. (2019). Sex differences in heart failure. Eur. Heart J..

[60] Pizzino G., Bitto A., Crea P., Khandheria B., Vriz O., Carerj S., Squadrito F., Minisini R., Citro R., Cusmà-Piccione M. (2017). Takotsubo syndrome and estrogen receptor genes: Partners in crime?. J Cardiovasc. Med..

[61] Crea P., Carerj S., Bitto A., Cusma-Piccione M., Vriz O., Madaffari A., Minisini R., Acri E., Oteri A., Zito C. (2013). Study of estrogen receptors polymorphisms in women with Takotsubo cardiomyopathy or myocardial infarction. Eur. Heart J..

[62] Münzel T., Templin C., Cammann V.L., Hahad O. (2021). Takotsubo Syndrome: Impact of endothelial dysfunction and oxidative stress. Free Radic. Biol. Med..

[63] Vendrov A.E., Xiao H., Lozhkin A., Hayami T., Hu G., Brody M.J., Sadoshima J., Zhang Y.Y., Runge M.S., Madamanchi N.R. (2023). Cardiomyocyte NOX4 regulates resident macrophage-mediated inflammation and diastolic dysfunction in stress cardiomyopathy. Redox Biol..

[64] Xiao H., Li H., Wang J.J., Zhang J.S., Shen J., An X.B., Zhang C.C., Wu J.M., Song Y., Wang X.Y. (2018). IL-18 cleavage triggers cardiac inflammation and fibrosis upon β-adrenergic insult. Eur. Heart J..

[65] Nef H.M., Möllmann H., Kostin S., Troidl C., Voss S., Weber M., Dill T., Rolf A., Brandt R., Hamm C.W. (2007). Tako-Tsubo cardiomyopathy: Intraindividual structural analysis in the acute phase and after functional recovery. Eur. Heart J..

[66] Gomez Perdiguero E., Klapproth K., Schulz C., Busch K., Azzoni E., Crozet L., Garner H., Trouillet C., de Bruijn M.F., Geissmann F. (2015). Tissue-resident macrophages originate from yolk-sac-derived erythro-myeloid progenitors. Nature.

[67] Liao X., Chang E., Tang X., Watanabe I., Zhang R., Jeong H.W., Adams R.H., Jain M.K. (2022). Cardiac macrophages regulate isoproterenol-induced Takotsubo-like cardiomyopathy. JCI Insight.

[68] Gong T., Liu L., Jiang W., Zhou R. (2020). DAMP-sensing receptors in sterile inflammation and inflammatory diseases. Nat. Rev. Immunol..

[69] King K.R., Aguirre A.D., Ye Y.X., Sun Y., Roh J.D., Ng R.P., Kohler R.H., Arlauckas S.P., Iwamoto Y., Savol A. (2017). IRF3 and type I interferons fuel a fatal response to myocardial infarction. Nat. Med..

[70] Wang Y., Tang X., Cui J., Wang P., Yang Q., Chen Y., Zhang T. (2024). Ginsenoside Rb1 mitigates acute catecholamine surge-induced myocardial injuries in part by suppressing STING-mediated macrophage activation. Biomed. Pharmacother..

[71] Behzadi P., García-Perdomo H.A., Karpiński T.M. (2021). Toll-Like Receptors: General Molecular and Structural Biology. J. Immunol. Res..

[72] Zhang Y., Wu J., Dong E., Wang Z., Xiao H. (2023). Toll-like receptors in cardiac hypertrophy. Front. Cardiovasc. Med..

[73] Kołodzińska A., Czarzasta K., Szczepankiewicz B., Główczyńska R., Fojt A., Ilczuk T., Budnik M., Krasuski K., Folta M., Cudnoch-Jędrzejewska A. (2018). Toll-like receptor expression and apoptosis morphological patterns in female rat hearts with takotsubo syndrome induced by isoprenaline. Life Sci..

[74] Uchida Y., Egami H., Uchida Y., Sakurai T., Kanai M., Shirai S., Nakagawa O., Oshima T. (2010). Possible participation of endothelial cell apoptosis of coronary microvessels in the genesis of Takotsubo cardiomyopathy. Clin. Cardiol..

[75] Riad A., zu Schwabedissen H.M., Weitmann K., Herda L.R., Dörr M., Empen K., Kieback A., Hummel A., Reinthaler M., Grube M. (2012). Variants of Toll-like receptor 4 predict cardiac recovery in patients with dilated cardiomyopathy. J. Biol. Chem..

[76] Incalcaterra E., Caruso M., Balistreri C.R., Candore G., Lo Presti R., Hoffmann E., Caimi G. (2010). Role of genetic polymorphisms in myocardial infarction at young age. Clin. Hemorheol. Microcirc..

[77] Hussain S., Jha S., Berger E., Molander L., Sevastianova V., Sheybani Z., Espinosa A.S., Elmahdy A., Al-Awar A., Kakaei Y. (2024). Comparative Analysis of Plasma Protein Dynamics in Women with ST-Elevation Myocardial Infarction and Takotsubo Syndrome. Cells.

[78] Nagai M., Shityakov S., Smetak M., Hunkler H.J., Bär C., Schlegel N., Thum T., Förster C.Y. (2023). Blood Biomarkers in Takotsubo Syndrome Point to an Emerging Role for Inflammaging in Endothelial Pathophysiology. Biomolecules.

[79] Di Gangi P., Scola L., Giambanco S., Bova M., Santini G., Vaccarino L., Balistreri C.R., Lio D., Novo G. (2014). Cytokine Polymorphism in Takotsubo Cardiomyopathy. Am. J. Pathol..

[80] O’Reardon J.P., Lott J.P., Akhtar U.W., Cristancho P., Weiss D., Jones N. (2008). Acute coronary syndrome (Takotsubo cardiomyopathy) following electroconvulsive therapy in the absence of significant coronary artery disease: Case report and review of the literature. J. ECT.

[81] Gargoum A., Bare I., Pekrul C., Nosib S. (2021). Loeys-Dietz syndrome and isolated severe ostial left main coronary stenosis presenting as ventricular fibrillation arrest and biventricular takotsubo syndrome in a 25-year-old patient. BMJ Case Rep..

[82] Scola L., Di Maggio F.M., Vaccarino L., Bova M., Forte G.I., Pisano C., Candore G., Colonna-Romano G., Lio D., Ruvolo G. (2014). Role of TGF-β pathway polymorphisms in sporadic thoracic aortic aneurysm: rs900 TGF-β2 is a marker of differential gender susceptibility. Mediat. Inflamm..

[83] Ueyama T., Kawabe T., Hano T., Tsuruo Y., Ueda K., Ichinose M., Kimura H., Yoshida K. (2009). Upregulation of heme oxygenase-1 in an animal model of Takotsubo cardiomyopathy. Circ. J..

[84] Willis B.C., Salazar-Cantú A., Silva-Platas C., Fernández-Sada E., Villegas C.A., Rios-Argaiz E., González-Serrano P., Sánchez L.A., Guerrero-Beltrán C.E., García N. (2015). Impaired oxidative metabolism and calcium mishandling underlie cardiac dysfunction in a rat model of post-acute isoproterenol-induced cardiomyopathy. Am. J. Physiol. Heart Circ. Physiol..

[85] Tsolaki V., Makris D., Mantzarlis K., Zakynthinos E. (2017). Sepsis-Induced Cardiomyopathy: Oxidative Implications in the Initiation and Resolution of the Damage. Oxid. Med. Cell. Longev..

[86] Nef H.M., Möllmann H., Troidl C., Kostin S., Böttger T., Voss S., Hilpert P., Krause N., Weber M., Rolf A. (2008). Expression profiling of cardiac genes in Tako-Tsubo cardiomyopathy: Insight into a new cardiac entity. J. Mol. Cell. Cardiol..

[87] Surikow S.Y., Nguyen T.H., Stafford I., Chapman M., Chacko S., Singh K., Licari G., Raman B., Kelly D.J., Zhang Y. (2018). Nitrosative Stress as a Modulator of Inflammatory Change in a Model of Takotsubo Syndrome. JACC Basic Transl. Sci..

[88] He W., Wang Q., Gu L., Zhong L., Liu D. (2018). NOX4 rs11018628 polymorphism associates with a decreased risk and better short-term recovery of ischemic stroke. Exp. Ther. Med..

[89] Theccanat T., Philip J.L., Razzaque A.M., Ludmer N., Li J., Xu X., Akhter S.A. (2016). Regulation of cellular oxidative stress and apoptosis by G protein-coupled receptor kinase-2; The role of NADPH oxidase 4. Cell. Signal..

[90] Lange L.A., Croteau-Chonka D.C., Marvelle A.F., Qin L., Gaulton K.J., Kuzawa C.W., McDade T.W., Wang Y., Li Y., Levy S. (2010). Genome-wide association study of homocysteine levels in Filipinos provides evidence for CPS1 in women and a stronger MTHFR effect in young adults. Hum. Mol. Genet..

[91] Wang S., Nikamo P., Laasonen L., Gudbjornsson B., Ejstrup L., Iversen L., Lindqvist U., Alm J.J., Eisfeldt J., Zheng X. (2024). Rare coding variants in NOX4 link high ROS levels to psoriatic arthritis mutilans. EMBO Mol. Med..

[92] Sato T., Hanna P., Mori S. (2024). Innervation of the coronary arteries and its role in controlling microvascular resistance. J. Cardiol..

[93] Schnabel R.B., Hasenfuß G., Buchmann S., Kahl K.G., Aeschbacher S., Osswald S., Angermann C.E. (2021). Heart and brain interactions: Pathophysiology and management of cardio-psycho-neurological disorders. Herz.

[94] Couch L.S., Fiedler J., Chick G., Clayton R., Dries E., Wienecke L.M., Fu L., Fourre J., Pandey P., Derda A.A. (2022). Circulating microRNAs predispose to takotsubo syndrome following high-dose adrenaline exposure. Cardiovasc. Res..

[95] Cho Y.Y., Kim S., Kim P., Jo M.J., Park S.E., Choi Y., Jung S.M., Kang H.J. (2025). G-Protein-Coupled Receptor (GPCR) Signaling and Pharmacology in Metabolism: Physiology, Mechanisms, and Therapeutic Potential. Biomolecules.

[96] Wieland T., Lutz S., Chidiac P. (2007). Regulators of G protein signalling: A spotlight on emerging functions in the cardiovascular system. Curr. Opin. Pharmacol..

[97] Kuwabara Y., Ono K., Horie T., Nishi H., Nagao K., Kinoshita M., Watanabe S., Baba O., Kojima Y., Shizuta S. (2011). Increased microRNA-1 and microRNA-133a levels in serum of patients with cardiovascular disease indicate myocardial damage. Circ. Cardiovasc. Genet..

[98] d’Avenia M., Citro R., De Marco M., Veronese A., Rosati A., Visone R., Leptidis S., Philippen L., Vitale G., Cavallo A. (2015). A novel miR-371a-5p-mediated pathway, leading to BAG3 upregulation in cardiomyocytes in response to epinephrine, is lost in Takotsubo cardiomyopathy. Cell Death Dis..

[99] Citro R., d’Avenia M., De Marco M., Giudice R., Mirra M., Ravera A., Silverio A., Farina R., Silvestri F., Gravina P. (2013). Polymorphisms of the antiapoptotic protein bag3 may play a role in the pathogenesis of tako-tsubo cardiomyopathy. Int. J. Cardiol..

[100] Gaddam R.R., Amalkar V.S., Sali V.K., Nakuluri K., Jacobs J.S., Kim Y.R., Li Q., Bahal R., Irani K., Vikram A. (2023). Role of miR-204 in segmental cardiac effects of phenylephrine and pressure overload. Biochem. Biophys. Res. Commun..

[101] Ibanez B., Benezet-Mazuecos J., Navarro F., Farre J. (2006). Takotsubo syndrome: A Bayesian approach to interpreting its pathogenesis. Mayo Clin. Proc..

[102] Haghi D., Roehm S., Hamm K., Harder N., Suselbeck T., Borggrefe M., Papavassiliu T. (2010). Takotsubo cardiomyopathy is not due to plaque rupture: An intravascular ultrasound study. Clin. Cardiol..

[103] Cecchi E., Parodi G., Fatucchi S., Angelotti P., Giglioli C., Gori A.M., Bandinelli B., Bellandi B., Sticchi E., Romagnuolo I. (2016). Prevalence of thrombophilic disorders in takotsubo patients: The (ThROmbophylia in TAkotsubo cardiomyopathy) TROTA study. Clin. Res. Cardiol..

[104] Santoro F., Tarantino N., Ieva R., Musaico F., Di Biase M., Brunetti N.D. (2014). Hereditary hypercoagulable state and Takotsubo cardiomyopathy: A possible link. Int. J. Cardiol..

[105] Bova M., Novo G., Scola L., Santini G., Di Gangi P., Lio D. Analysis of coagulation gene SNPs support the role of acute phase reaction and catecholamine excess in takotsubo cardiomyopathy. Proceedings of the 1st SIPMeL National Congress—La Medicina di Laboratorio Guarda al Future.

[106] Del Buono M.G., Montone R.A., Camilli M., Carbone S., Narula J., Lavie C.J., Niccoli G., Crea F. (2021). Coronary Microvascular Dysfunction Across the Spectrum of Cardiovascular Diseases: JACC State-of-the-Art Review. J. Am. Coll. Cardiol..

[107] Flammer A.J., Lüscher T.F. (2010). Human endothelial dysfunction: EDRFs. Pflug. Arch..

[108] Yang Z., Li Y., Huang M., Li X., Fan X., Yan C., Meng Z., Liao B., Hamdani N., El-Battrawy I. (2024). Small conductance calcium-activated potassium channel contributes to stress induced endothelial dysfunctions. Microvasc. Res..

[109] Kalani M.Y.S., Siniard A.L., Corneveaux J.J., Bruhns R., Richholt R., Forseth J., Zabramski J.M., Nakaji P., Spetzler R.F., Huentelman M.J. (2016). Rare Variants in Cardiomyopathy Genes Associated with Stress-Induced Cardiomyopathy. Neurosurgery.

[110] d’Apolito M., Santoro F., Ranaldi A., Cannito S., Santacroce R., Ragnatela I., Margaglione A., D’Andrea G., Brunetti N.D., Margaglione M. (2025). Genetic Background and Clinical Phenotype in an Italian Cohort with Inherited Arrhythmia Syndromes and Arrhythmogenic Cardiomyopathy (ACM): A Whole-Exome Sequencing Study. Int. J. Mol. Sci..

[111] Frederiksen T.C., Calloe K., Geryk M., Jensen H.K. (2022). Takotsubo cardiomyopathy and Brugada syndrome in a patient with a novel loss-of-function variant in the cardiac sodium channel Na_v_1.5. HeartRhythm Case Rep..

[112] Eitel I., Moeller C., Munz M., Stiermaier T., Meitinger T., Thiele H., Erdmann J. (2017). Genome-wide association study in takotsubo syndrome—Preliminary results and future directions. Int. J. Cardiol..

[113] Dattilo V., Ulivi S., Minelli A., La Bianca M., Giacopuzzi E., Bortolomasi M., Bignotti S., Gennarelli M., Gasparini P., Concas M.P. (2023). Genome-wide association studies on Northern Italy isolated populations provide further support concerning genetic susceptibility for major depressive disorder. World J. Biol. Psychiatry.

[114] Zorn P., Calvo Sánchez J., Alakhras T., Schreier B., Gekle M., Hüttelmaier S., Köhn M. (2024). Rbfox1 controls alternative splicing of focal adhesion genes in cardiac muscle cells. J. Mol. Cell. Biol..

[115] Zeng Q., Cai J., Wan H., Zhao S., Tan Y., Zhang C., Qu S. (2021). PIWI-interacting RNAs and PIWI proteins in diabetes and cardiovascular disease: Molecular pathogenesis and role as biomarkers. Clin. Chim. Acta.

[116] Yu Y., Xia L.K., Di Y., Nie Q.Z., Chen X.L. (2023). Mechanism of piR-1245/PIWI-like protein-2 regulating Janus kinase-2/signal transducer and activator of transcription-3/vascular endothelial growth factor signaling pathway in retinal neovascularization. Neural Regen Res..

[117] Verna E., Provasoli S., Ghiringhelli S., Morandi F., Salerno-Uriarte J. (2018). Abnormal coronary vasoreactivity in transient left ventricular apical ballooning (tako-tsubo) syndrome. Int. J. Cardiol..

[118] Patel S.M., Lerman A., Lennon R.J., Prasad A. (2013). Impaired coronary microvascular reactivity in women with apical ballooning syndrome (Takotsubo/stress cardiomyopathy). Eur. Heart J. Acute Cardiovasc. Care.

[119] Wong W.M., Mahroo O.A. (2025). Monogenic Retinal Diseases Associated with Genes Encoding Phototransduction Proteins: A Review. Clin. Exp. Ophthalmol..

[120] Li L., Zhao H., Xie H., Akhtar T., Yao Y., Cai Y., Dong K., Gu Y., Bao J., Chen J. (2021). Electrophysiological characterization of photoreceptor-like cells in human inducible pluripotent stem cell-derived retinal organoids during in vitro maturation. Stem Cells.

[121] Hamm M.J., Kirchmaier B.C., Herzog W. (2016). Sema3d controls collective endothelial cell migration by distinct mechanisms via Nrp1 and PlxnD1. J. Cell Biol..

[122] Corà D., Astanina E., Giraudo E., Bussolino F. (2014). Semaphorins in cardiovascular medicine. Trends Mol. Med..

[123] Aghajanian H., Cho Y.K., Manderfield L.J., Herling M.R., Gupta M., Ho V.C., Li L., Degenhardt K., Aharonov A., Tzahor E. (2016). Coronary vasculature patterning requires a novel endothelial ErbB2 holoreceptor. Nat. Commun..

[124] Santamaria S. (2020). ADAMTS-5: A difficult teenager turning 20. Int. J. Exp. Pathol..

[125] Hemmeryckx B., Carai P., Lijnen R.H. (2019). ADAMTS5 deficiency in mice does not affect cardiac function. Cell. Biol. Int..

[126] Suna G., Wojakowski W., Lynch M., Barallobre-Barreiro J., Yin X., Mayr U., Baig F., Lu R., Fava M., Hayward R. (2018). Extracellular Matrix Proteomics Reveals Interplay of Aggrecan and Aggrecanases in Vascular Remodeling of Stented Coronary Arteries. Circulation.

[127] Alabi R.O., Farber G., Blobel C.P. (2018). Intriguing Roles for Endothelial ADAM10/Notch Signaling in the Development of Organ-Specific Vascular Beds. Physiol. Rev..

[128] Xu T., Jiang S., Liu T., Han S., Wang Y. (2024). ADAM10 Alleviates Hypoxia/Reoxygenation-Induced Cardiomyocyte Injury by Activating the Notch Signaling Pathway. Cell Biochem. Biophys..

[129] Zaker S.R., Ghaedi K. (2021). Downregulation of LINC02615 is correlated with the breast cancer progress: A novel biomarker for differential identification of breast cancer tissues. Cell J..

[130] Yang C., Wu J., Lu X., Xiong S., Xu X. (2022). Identification of novel biomarkers for intracerebral hemorrhage via long noncoding RNA-associated competing endogenous RNA network. Mol. Omics.

[131] Saeidi L., Ghaedi H., Sadatamini M., Vahabpour R., Rahimipour A., Shanaki M., Mansoori Z., Kazerouni F. (2018). Long non-coding RNA LY86-AS1 and HCG27_201 expression in type 2 diabetes mellitus. Mol. Biol. Rep..

[132] Hakansson H., Tornvall P. (2024). A Genome-Wide Association Study (GWAS) of Takotsubo syndrome. Eur. Heart J..

[133] Li C., Zhang K., Zhao J. (2025). Genome-wide Mendelian randomization mapping the influence of plasma proteome on major depressive disorder. J. Affect. Disord..

[134] Garshick M.S., Barrett T.J., Cornwell M.G., Drenkova K., Garelik J., Weber B.N., Schlamp F., Rockman C., Ruggles K.V., Reynolds H.R. (2023). An inflammatory transcriptomic signature in psoriasis associates with future cardiovascular events. J. Eur. Acad. Dermatol. Venereol..

